# Preliminary evaluation of the Chinese version of the patient-reported outcomes measurement information system 29-item profile in patients with aortic dissection

**DOI:** 10.1186/s12955-022-02000-1

**Published:** 2022-06-14

**Authors:** Wanbing Huang, Qiansheng Wu, Yufen Zhang, Chong Tian, Haishan Huang, Sufang Huang, Yanrong Zhou, Jing He, Hui Wang

**Affiliations:** 1 Department of Nursing, Tongji Hospital, Tongji Medical College, Huazhong University of Science and Technology, Jie Fang Avenue 1095, Qiaokou District, Wuhan, 430030 China; 2 School of Nursing, Tongji Medical College, Huazhong University of Science and Technology, Hangkong Road 13, Qiaokou District, Wuhan, 430030 China; 3 Division of Cardiothoracic and Vascular Surgery, Tongji Hospital, Tongji Medical College, Huazhong University of Science and Technology, Jie Fang Avenue 1095, Qiaokou District, Wuhan, 430030 China; 4 Department of Emergency, Tongji Hospital, Tongji Medical College, Huazhong University of Science and Technology, Jie Fang Avenue 1095, Qiaokou District, Wuhan, 430030 China

**Keywords:** PROMIS-29, Patient reported outcome, Aortic dissection, Validity, Reliability

## Abstract

**Background:**

The Patient-Reported Outcomes Measurement Information System 29-item Profile (PROMIS-29) has been widely used to measure health outcomes from the patient’s perspective. It has not been validated in adults with aortic disease. The aim of this study was to explore the reliability and validity of the Chinese PROMIS-29 among patients undergoing surgery for aortic dissection (AD).

**Methods:**

A cross-sectional design was applied. Eligible patients completed a questionnaire that contained the PROMIS-29 and legacy measures, including the Short Form-12 Health Survey (SF-12), 8-item Somatic Symptom Scale (SSS-8), Generalized Anxiety Disorder–2 (GAD-2), and Patient Health Questionnaire-2 (PHQ-2). The structural validity of the PROMIS-29 was evaluated using confirmatory factor analysis (CFA). Reliability was evaluated with Cronbach’s α. Construct validity was assessed by calculating Spearman’s rank correlations and comparing known-group differences.

**Results:**

In total, a sample of 327 AD patients was included in the final analysis. Most of them were male (89%) with a mean age of 52.7 (± 10.3). CFA revealed good model fit of the seven-factor structure within PROMIS-29, as well as most domains in single-factor analysis. Reliability was confirmed with Cronbach’s α > 0.90. Correlations between comparable domains of the PROMIS-29 and those of legacy questionnaires and most know-group comparisons were observed as hypothesized.

**Conclusions:**

This study found evidence for acceptable structural validity, construct validity and internal consistency of the PROMIS-29 in a sample of AD patients. It can be applied to AD survivors by researchers or clinicians, measuring outcomes after surgery and identifying those with worse health status.

## Background

Aortic dissection (AD) is a relatively rare vascular disease due to an intimal tear in the inner layer of the aorta, which leads to separation of the aortic wall [1]. Since the hemodynamic stability of the whole body is seriously affected, AD is a much-feared clinical presentation that has a high mortality [2]. Surgery is the first choice for the treatment of AD, usually including thoracotomy based on cardiopulmonary bypass and endovascular repair. Studies have shown that timely surgery can significantly reduce AD mortality [3]. The survival rate of AD patients undergoing surgical treatment is more than 80% at 5 years [4, 5].

For a long time, AD mortality rates and adverse events have been widely published as key indicators for evaluating surgical outcomes [6–8]. Patient reported outcomes (PROs) are emerging as an important component for patient care in cardiovascular diseases and have been reported to be critical as clinical assessments in the evaluation of treatment outcomes [9]. However, there is little information about PROs among AD patients who underwent surgery. Evidence suggests that AD survivors may still experience disorders such as pain, fatigue, anxiety, depression, sleep problems and limitations in physical activity during long-term recovery after surgery [10–12]. Accurate measurements of such PROs will provide essential information to guide AD patient health education and self-management but remain to be further investigated.

In recent years, the Patient Reported Outcomes Measurement Information System (PROMIS^®^) has drawn great interest as a new and efficient instrument to evaluate person-centered health. It is a set of measures assessing physical, mental, and social health status, developed by a cooperative group of scientists from several academic institutions based on support of the United States (US) National Institutes of Health. PROMIS item banks allow universal assessment of symptoms and functions, enabling comparison of patient-reported outcomes across a spectrum of chronic conditions [13], and have been validated in extensive community and clinical samples [14, 15]. One of the most commonly used PROMIS measures is the PROMIS-29 profile. This measure is widely practiced worldwide and is already available in more than 40 languages. Due to its brevity and breadth, the PROMIS-29 has been examined in a broad range of general [16] or patient populations, such as adults with hemophilia [17], chronic low back pain [18], chronic pulmonary diseases [19], burn survivors [20] and kidney transplant recipients [21]. Good and robust psychometric properties have been established within these different settings.

However, research on the use of PROMIS tools in cardiovascular patients is very limited; in addition, the PROMIS-29 has not been validated or applied in the AD patient population. It measures seven domains, including physical function, anxiety, depression, fatigue, sleep disturbance, ability to participate in social roles and activities, and pain interference. These domains embrace the most frequently reported health and function problems among AD survivors, which may offer a comprehensive evaluation of a patient’s self-rated health status. The PROMIS-29 has been translated into Chinese by the main author and colleagues from the PROMIS Health Organization. There is already evidence that the Chinese version of the PROMIS profiles have sufficient linguistic equivalence and cross-cultural validity [22–24]. Therefore, the purpose of this study is to preliminarily verify the reliability and validity of the Chinese PROMIS-29 in the AD population.

## Methods

### Study populations and procedure

This was a single-center, cross-sectional study. The recruited participants were discharged AD patients undergoing surgery from the cardiac surgical department of a large public hospital in Wuhan City, China. The selected date of surgery was from January 2019 to May 2021.

Patients were eligible for the study if they met the following criteria: (1) Over 18 years old; (2) The presence of AD, confirmed by computed tomography angiography scans; and (3) Underwent thoracotomy or endovascular repair surgery. (4) Being able to speak Mandarin and read Chinese.

The exclusion criteria were as follows: (1) Intramural hematoma or aneurysm; (2) Patients had Marfan syndrome or Ehlers-Danlos syndrome; (3) AD secondary to trauma, iatrogenic injury, or pregnancy; and (4) A history of malignant tumors or cognitive impairment.

At least 2 months after surgery, eligible patients were approached via telephone contact, and verbal consent was obtained before data collection. Participants were asked to fill in their responses to all items in the PROMIS-29 and legacy measures through an online questionnaire shared by sending messages. Alternatively, patients were given another option to receive a telephone interview. Data on every respondent were collected only once. These surveys were conducted from July 2021 through December 2021. Approval was obtained from the Medical Ethical Committee of Tongji Medical College, Huazhong University of Science and Technology (registration number 2021S122).

### Measures

#### PROMIS-29

The Chinese version of the PROMIS-29 Profile v2.1 was used in this study. Items of this profile had been translated from English into Simplified Chinese by the main author and qualified translators in cooperation with the Director of Translations for PROMIS and approved according to rigorous standards presented by the PROMIS Health Organization. Permission was obtained on June 30, 2021.

The PROMIS-29 consists of 28 items measuring seven health and function domains: physical function, anxiety, depression, fatigue, sleep disturbance, ability to participate in social roles and activities, pain interference, with 4 items each, and an additional single item about pain intensity. Items were rated on a 5-point Likert-type scale from 1 to 5, and higher scores represent a higher degree of the trait being measured. In addition, pain intensity was assessed with a numeric rating scale from 0 to 10. The PROMIS-29 domain scores were calculated as T-scores through the online Assessment Center Scoring Service (https://www.assessmentcenter.net/). The T-Score is a metric with a mean of 50 and a standard deviation (SD) of 10 in the US general population. For physical function and social role, higher scores indicate better functioning and quality of life (QOL). For depression, anxiety, fatigue, pain interference, pain intensity, and sleep disturbance, a higher score indicates more serious implications of disease.

#### Legacy measures

The Short Form-12 Health Survey (SF-12) is a widely used generic measure of health status with established psychometric validity. The SF-12 has 12 items that are included in the Short Form Health Survey (SF-36) of the Medical Outcomes Study, assessing 8 dimensions (physical functioning (PF), role limitations due to physical health problems, bodily pain, general health, vitality, social functioning (SF), role limitations due to emotional problems and mental health). Scores can be summarized into a physical component summary (PCS) and mental component summary (MCS) [25], with an average score of 50 and an SD of 10 in the reference population (the US general population) [26]. Higher scores represent better health.

The 8-item Somatic Symptom Scale (SSS-8) is a short and valid patient-reported measure of somatic symptom burden. The 8 items in the SSS-8 are a subset of those in the Patient Health Questionnaire-15 (PHQ-15) [27]. It assesses 8 common symptoms, including bowel problems, back pain, pain in arms, legs, or joints, headaches, chest pain or shortness of breath, dizziness, tiredness and trouble sleeping. The 7-day time frame in SSS-8 items was similar to those in PROMIS-29. A 5-point response scale (0–4) for each SSS-8 item was used; therefore, the total score ranged from 0 to 32, which can be classified into five somatic symptom severities: 0 to 3 as none to minimal, 4 to 7 as low, 8 to 11 as medium, 12 to 15 as high, and 16 to 32 as very high levels of physical symptoms [28]. The SSS-8 was a reference instrument in the Fifth Diagnostic and Statistical Manual of Mental Disorders (DSM-5) field trials [29] and its validity and internal consistency have been confirmed in various cultural contexts. Recently, it has been increasingly used as a promising tool for the rapid recognition of symptom burden in the Chinese population [30, 31].

To evaluate the construct validity of the PROMIS-29, the PHQ-2 depression scale and the Generalized Anxiety Disorder-2 (GAD-2) scale were also administered. These two scales contain the first two items from the PHQ-9 and GAD-7 [32]. The PHQ-2 and GAD-2 are brief tools to assess the presence and severity of depressive or anxiety symptoms, with great reliability and validity in the Chinese population [33, 34]. Items in the PHQ-2 and GAD-2 are both rated on a 4-poin scale (0–3) and range from 0 to 6. A higher score reflects a higher degree of depression or anxiety.

Sociodemographic and clinical characteristics, such as age, sex, educational level, and marital status, were also included in the questionnaire. Clinical characteristics were collected from electronic medical records, and the Charlson Comorbidity Index (CCI) was calculated to define the presence of comorbidity.

#### Statistical analysis

Descriptive statistics, including the mean, standard deviation (SD) and median, were used to determine the sample characteristics and distribution of domain scores. Floor or ceiling effects are defined as the proportion of patients scoring the minimum (floor) or maximum (ceiling) possible score for each domain. When either the proportion of minimum or maximum response was greater than 20% [35], floor or ceiling effects were considered noteworthy, respectively.

Confirmatory factor analysis (CFA) was carried out using maximum likelihood estimation to examine the structural validity of the PROMIS domains. The fitness of the proposed 7-factor model to the data was evaluated using the comparative fit index (CFI) and standardized root-mean-squared residual (SRMR). A CFI > 0.95 and SRMR < 0.05 indicate a good fit. Other indicators included the root mean square error of approximation (RMSEA) and Akaike’s information criterion (AIC). RMSEA < 0.05 indicates a good fit, and < 0.08 is also acceptable [36]. The smaller the AIC value is, the better the model fit. Furthermore, a single-factor CFA equation for each domain was also tested separately, and the criteria used were as stated above. To evaluate reliability, Cronbach’s α coefficients were used to calculate the internal consistency of each domain of the PROMIS-29. An α value of ≥ 0.70 was considered satisfactory [37].

To evaluate convergent validity, we calculated Spearman’s rank correlations between scores of PROMIS-29 and their corresponding legacy PRO measures as follows: PROMIS-29 physical function and SF-12 PF or SF-12 PCS, PROMIS-29 anxiety and GAD-2 or SF-12 MCS, PROMIS-29 depression and PHQ-2 or SF-12 MCS, PROMIS-29 fatigue and SF-12 Vitality or SSS-8 tiredness item, PROMIS-29 sleep disturbance and SSS-8 trouble sleeping item, PROMIS-29 ability to participate in social roles and activities and SF-12 SF, PROMIS-29 pain interference and SSS-8 Pain. The magnitude of the correlation coefficients was interpreted as high (r ≥ 0.7), moderate (r = 0.5–0.7) and low (r < 0.5) [38]. Based on published literature, the PROMIS-29 subscales were hypothesized to have high correlations (r ≥ 0.7) [17] between similar legacy PRO subscales (Table 5).

Discriminant validity was supported through comparing correlations between scores of PROMIS-29 domains and dissimilar constructs of legacy measures; these correlations were expected to be less than 0.60 [17]. The construct validity of the PROMIS-29 was also assessed by conducting a known-groups analysis. The mean T-scores between relevant sociodemographic and clinical groups were compared using the Mann–Whitney U test. These groups were defined based on a review of previous literature as well as the authors’ clinical experience. All hypothesized magnitudes of correlations and known-group differences are shown in Table 5. The criterion that at least 75% of the results should correspond with these hypotheses was used to determine the sufficient construct validity [39].

IBM SPSS Statistics software (version 20.0) was used to conduct statistical analyses. CFA was performed with IBM SPSS Amos Graphics (version 21.0). All significance tests were 2-tailed, with *p* < 0.05 considered significant.

## Results

### Participants characteristics

**Fig. 1:**
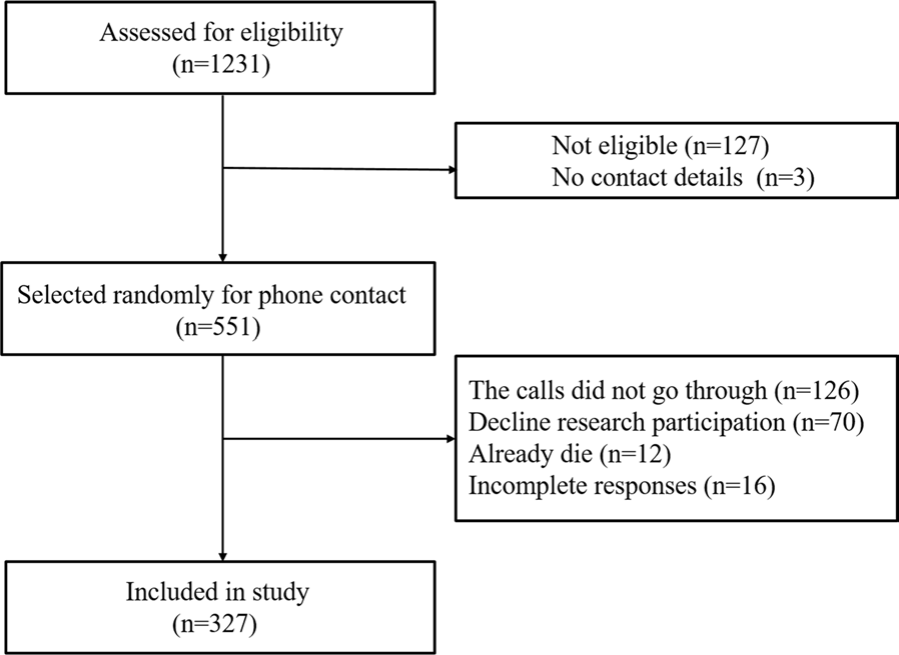
Patient selection flow chart

A total of 551 eligible patients were approached, and 343 patients consented to participate in this study (response rate 62.3%). After eliminating severely incomplete responses data, a sample of 327 was chosen for the final analysis (Fig. 1). Most patients were male (89%), with a mean age of 52.7 (± 10.3, range 24–80). The majority of participants were married (96.3%), and 41.6% were at or above high school or equivalent level education. Of all the participants, 68.8% were diagnosed with Stanford type B AD, and the mean follow-up was 11 ± 4.3 (range 2.6–21.6) months after surgery. Two hundred and eighty-three (86.5%) of the patients had hypertension. The median CCI was 1 (range 0–7), and 16.5% of the individuals were divided into a high comorbidity burden (CCI ≥ 3). See more detail in Table 1.

**Table 1:** characteristics of the study Patient (N = 327)

Characteristic		
Sex [n (%)]	Male	291 (89)
	Female	36 (11)
Age at the time of surgery, (mean ± SD)		52.7 ± 10.3
BMI (kg/m^2^) (mean ± SD)		25.8 ± 3.6
Education [n (%)]	Less than high school/ ≤ Middle school	191 (58.4)
	High school or equivalent	72 (22.0)
	College level or higher	64(19.6)
Marital status	Married	315 (96.3)
	Single, divorced, widowed	12 (3.7)
Type of AD	Stanford A	102 (31.2)
	Stanford B	225 (68.8)
Hypertension [n (%)]	Yes	283 (86.5)
	No	44(13.5)
Coronary heart disease	Yes	17 (5.2)
	No	310(94.8)
Diabetes mellitus	Yes	13 (4.0)
	No	314(96.0)
Arteriosclerosis	Yes	47 (14.4)
	No	280(85.6)
CCI score [n (%)]	0	108 (33.0)
	1	104 (31.8)
	2	61 (18.7)
	≥ 3	54 (16.5)
Operative procedure	Endovascular repair	231(70.6)
	Thoracotomy	96(29.4)
Months from surgery, (mean ± SD)		11 ± 4.3

### Description of measures

**Fig. 2:**
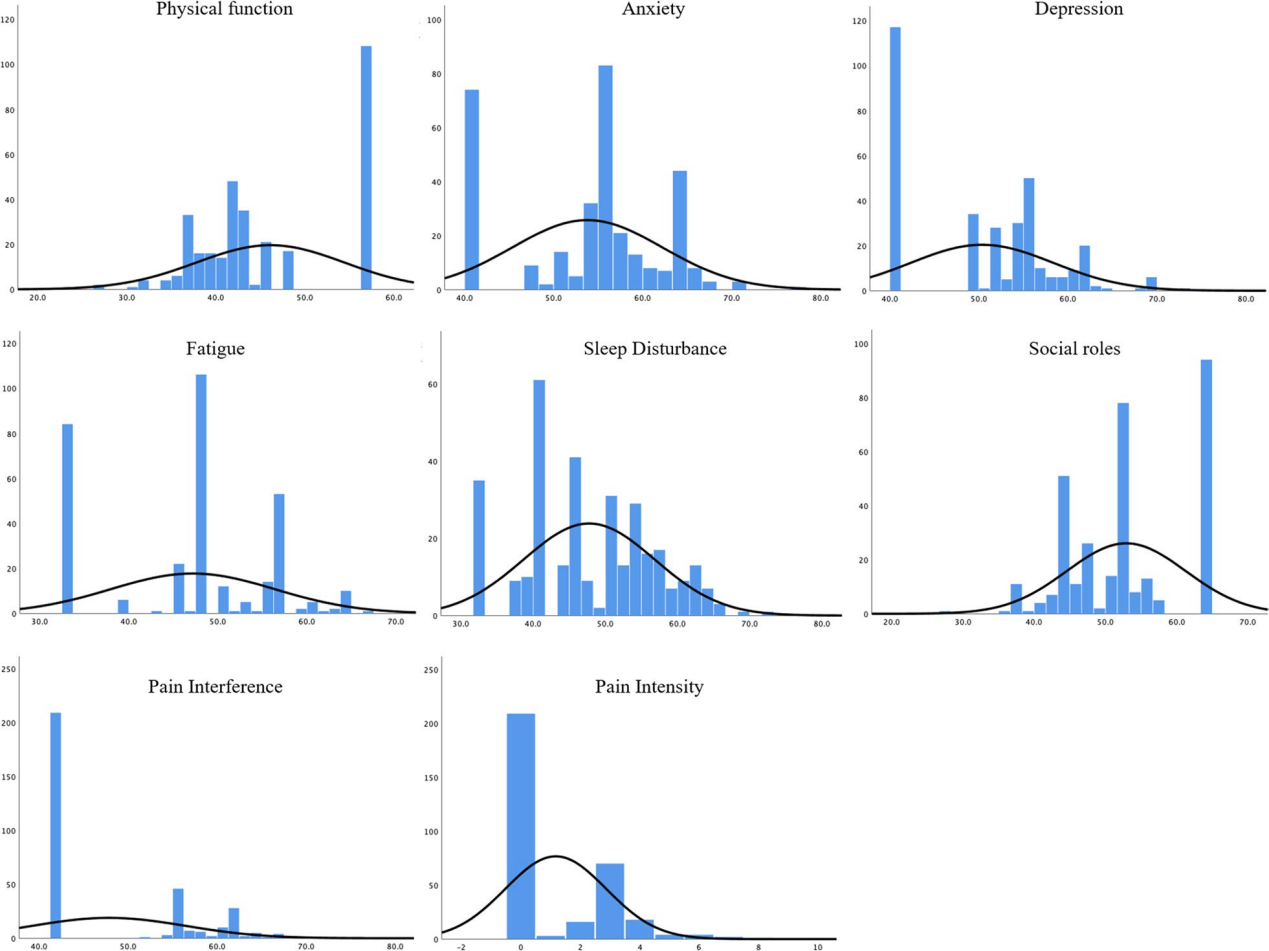
Distribution of T-scores for each domain and raw scores for pain intensity within the PROMIS-29

Descriptive statistics, including the mean and SDs for the PROMIS-29 and legacy measures, are presented in Table 2. The distribution was highly skewed for physical function, depression, ability to participate in social roles and activities, pain interference and pain intensity (Fig. 2). Compared to the US general population, this sample of patients had higher levels of anxiety (53.8 ± 8.4) and depression (50.4 ± 8.0) and worse physical function (46.1 ± 8.3).

**Table 2:** Scores and fit indices of CFA for PROMIS-29 domains (n = 327)

Model	Mean	SD	CFI	SRMR	RMSEA	AIC	Cranach’s α
Seven-factor model			0.97	0.03	0.06	918.38	
Single-factor model							
Physical function	46.1	8.3	0.95	0.03	**0.31**	79.02	0.95
Anxiety	53.8	8.4	0.99	0.01	0.04	18.97	0.95
Depression	50.4	8.0	0.98	0.02	**0.19**	41.16	0.93
Fatigue	47.2	9.2	0.99	0.01	**0.13**	28.93	0.97
Sleep Disturbance	47.8	9.1	**0.94**	0.04	**0.33**	89.92	0.92
Ability to participate in social roles and activities	52.9	8.3	0.97	0.02	**0.26**	62.59	0.97
Pain Interference	47.8	8.6	0.99	0.01	**0.21**	45.36	0.99

Bold = not meets cutoff

Floor and ceiling effects were observed for both the PROMIS-29 and other measures. Significant floor effects were seen on pain interference (63.9%), depression (35.8%), fatigue (25.7%), and anxiety (22.6%). Meanwhile, there were substantial ceiling effects on physical function (33.0%) and the ability to participate in social roles and activities (28.7%). Overall, floor effects and ceiling effects were found to be smaller for most PROMIS-29 domains than for the legacy measures (Table 3).

**Table 3:** The floor and ceiling effects and Spearman coefficients within PROMIS-29 scales and against legacy PRO domains

PROMIS-29	Floor (%)	Ceiling (%)	Physical function	Anxiety	Depression	Fatigue	Sleep disturbance	Social roles	Pain interference
Physical function	0.6	**33.0**		− 0.52	− 0.47	− 0.69	− 0.47	0.76	− 0.46
Anxiety	**22.6**	0.3	− 0.52		0.75	0.58	0.58	− 0.44	0.31
Depression	**35.8**	0.3	− 0.47	0.75		0.56	0.57	− 0.43	0.32
Fatigue	**25.7**	0.3	− 0.69	0.58	0.56		0.63	− 0.68	0.45
Sleep disturbance	10.7	0.3	− 0.47	0.58	0.57	0.63		− 0.48	0.32
Social roles	0.3	**28.7**	0.76	− 0.44	− 0.43	− 0.68	− 0.48		− 0.59
Pain interference	**63.9**	0.3	− 0.46	0.31	0.32	0.45	0.32	− 0.59	
SF-12 PCS			0.59	− 0.15	− 0.14	− 0.29	− 0.19	0.41	0.03
SF-12 MCS			0.06	− 0.52	− 0.30	− 0.12	− 0.17	0.05	0.00
SF-12 PF	8.0	**35.8**	0.90	− 0.51	− 0.43	− 0.63	− 0.45	0.70	− 0.36
GAD-2	**28.7**	0.9	− 0.49	0.85	0.64	0.51	0.51	− 0.42	0.27
PHQ-2	**40.4**	1.8	− 0.48	0.74	0.87	0.55	0.54	− 0.40	0.27
SF − 12 vitality	6.1	5.5	0.61	− 0.52	− 0.54	− 0.77	− 0.56	0.62	− 0.46
SSS-8 tiredness	**29.7**	0.3	− 0.68	0.58	0.56	0.91	0.6	− 0.66	0.39
SSS-8 sleep	**39.8**	0.9	− 0.46	0.60	0.55	0.61	0.91	− 0.46	0.26
SF-12 SF	8.0	**35.8**	0.60	− 0.44	− 0.39	− 0.51	− 0.39	0.71	− 0.52
SSS-8 pain	**69.7**	0.3	− 0.36	0.26	0.32	0.38	0.31	− 0.50	0.87

Bold = not meets cutoff

### Structural validity

**Fig. 3:**
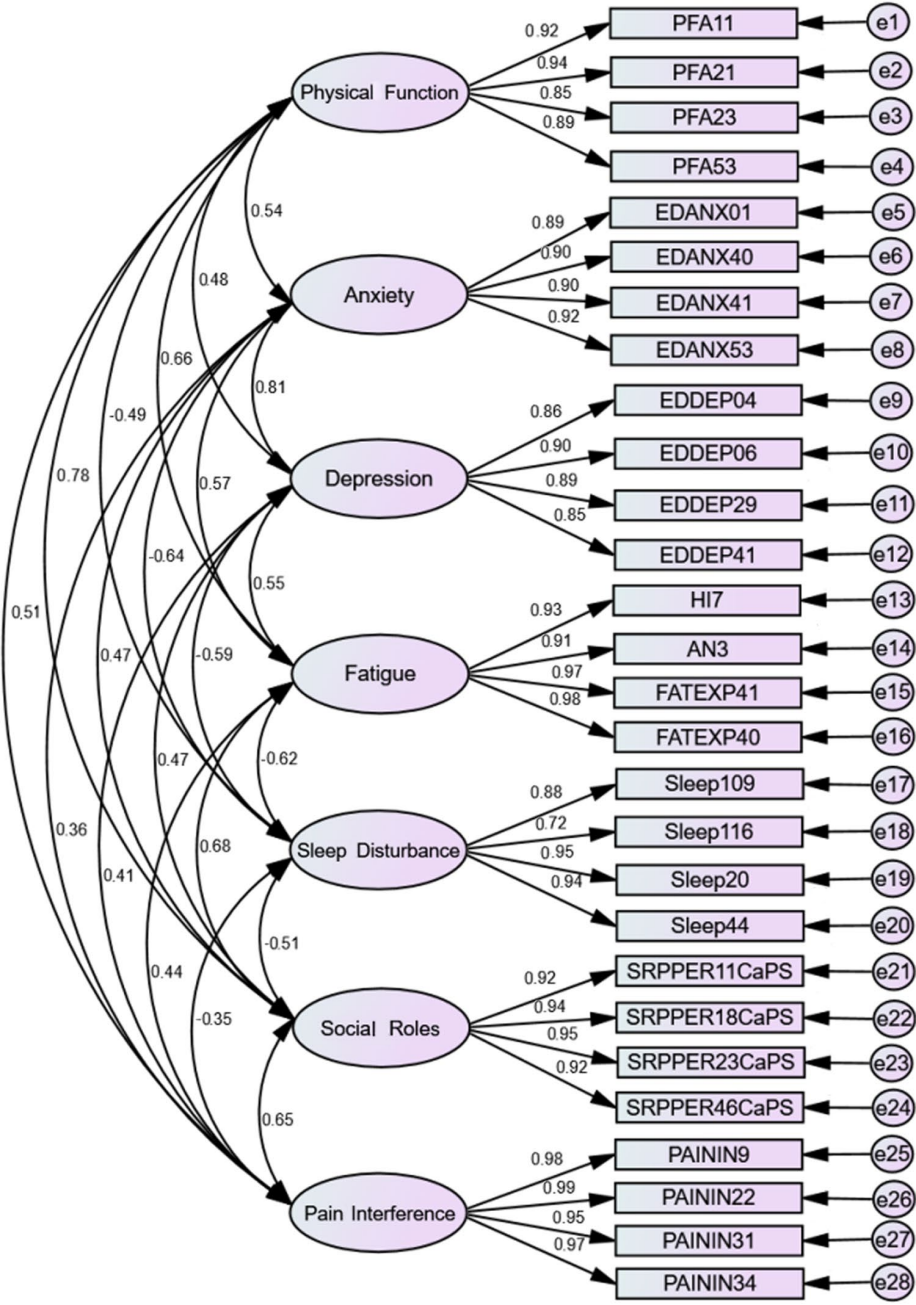
Confirmatory factor analysis for the seven-factor model of the PROMIS-29

The goodness-of-fit indices and main model fit results of CFA are shown in Table 2. The original seven-factor model structure within PROMIS-29 was confirmed based on all the statistics (CFI = 0.97, SRMR = 0.03, RMSEA = 0.06). More details are presented in Fig. 3. Further analysis revealed that the single-factor model demonstrated good model fit for most domains within PROMIS-29, with CFI ranging from 0.95 to 0.99 and SRMR ranging from 0.01 to 0.03 (Table 2). For sleep disturbance, the model fit might be less optimal, with a CFI of only 0.94 and a relatively larger AIC value, and error covariances were observed between items about “sleep quality” and “sleep was refreshing”. Notably, most scales failed to meet the RMSEA criteria of < 0.08, which was also seen in previous studies about PROMIS instruments [40, 41].

### Convergent validity and discriminant validity

The correlations between most PROMIS-29 domains and the comparable PRO measures were significantly strong (r > 0.70), as presented in Table 3. The largest correlation was found between sleep disturbance and the SSS-8 sleep item (r = 0.91). Convergent validity was thus considered to be achieved.

Satisfied discriminant validity was supported by the observation that correlations between PROMIS-29 domain scores and conceptually similar scales were stronger than divergent legacy scales. For instance, PROMIS-29 physical function score correlated with GAD-2 and PHQ-2 at a relatively low degree (r < 0.6), as well as between PROMIS-29 anxiety and depression and SF-12 PF. The inter-factor correlations were at a weaker level for most PROMIS-29 domains, as expected. See details in Table 3.

Known-group differences of PROMIS-29 T-scores are shown in Table 4. The female patients had a relatively lower physical function score (*p* = 0.008) and higher fatigue score (*p* = 0.002) than the male patients. Compared to patients below 50 years, older patients (> 65 years old) had a lower mean PROMIS physical function score (*p* < 0.001). Compared with patients with type A AD, type B patients had higher physical function scores (44.3 vs. 47.0, *p* = 0.021) and lower anxiety scores (53.4 vs. 54.7, *p* = 0.045). People with a high comorbidity burden (CCI ≥ 3) had a lower physical function score (*p* = 0.001) and a higher fatigue score (*p* = 0.037). Scores of ability to participate in social roles and activities were able to discriminate between predefined gender, age, type and CCI groups (*p* < 0.05).

**Table 4:** Known-Group Analyses Results of PROMIS-29 T-scores

T-scores	Gender [Mean(SD)]				Age [Mean(SD)]				Type [Mean(SD)]				CCI Score [Mean(SD)]			
	Male	Female	|d|	*p* value	Young (< 50)	Old(> 65)	|d|	*p* value	Type A	Type B	|d|	*p* value	CCI < 2	CCI ≥ 3	|d|	*p* value
Physical function	46.5 (8.3)	42.9 (7.7)	3.6	**0.008**	48.3 (8.4)	42.1 (7.3)	6.2	**0.000**	44.3 (8.2)	47.0 (8.3)	2.7	**0.021**	47.3 (8.3)	43.2 (7.5)	4.1	**0.001**
Anxiety	53.8 (8.3)	54.0 (9.9)	0.2	0.841	54.1 (8.9)	52.6 (8.1)	1.5	0.241	54.7 (9.1)	53.4 (8.1)	1.3	**0.045**	53.9 (8.6)	54.0 (7.9)	0.1	0.699
Depression	50.2 (7.8)	51.7 (9.3)	1.5	0.335	50.5 (8.4)	50.0 (6.6)	0.5	0.672	51.5 (8.7)	49.8 (7.6)	1.7	0.064	50.2 (8.4)	51.3 (7.0)	1.1	0.573
Fatigue	46.7 (9.2)	51.3 (8.9)	4.6	**0.002**	45.8 (9.6)	49.5 (9.4)	3.7	0.063	47.4 (9.6)	47.0 (9.0)	0.4	0.534	46.3 (9.2)	49.6 (9.2)	3.3	**0.037**
Sleep disturbance	47.5 (9.1)	49.7 (9.3)	2.2	0.200	47.1 (9.7)	49.8 (9.0)	2.7	0.192	48.9 (9.4)	47.2 (8.9)	1.7	0.074	47.2 (9.2)	49.6 (8.4)	2.4	0.086
Social roles	53.3 (8.3)	50.0 (8.2)	3.3	**0.030**	54.8 (8.4)	50.2 (8.4)	4.6	**0.003**	51.1 (8.5)	53.8 (8.2)	2.7	**0.026**	53.6 (8.4)	50.5 (7.6)	3.1	**0.009**
Pain interference	47.5 (8.4)	50.6 (9.7)	3.1	0.060	46.3 (7.9)	47.8 (9.1)	1.5	0.331	49.0 (9.3)	47.4 (8.3)	1.6	0.137	47.7 (8.6)	48.4 (8.5)	0.7	0.522

Bold = *p* value < 0.05; |d|: absolute value of mean T-scores differences

Overall, 69 of 84 hypotheses (82%) were confirmed. Six of the seven PROMIS-29 subscales were considered to have sufficient construct validity (≥ 75% hypotheses confirmed): physical function (76%), anxiety (82%), fatigue (82%), sleep disturbance (91%), ability to participate in social roles and activities (86%), and pain interference (91%). For the depression subscale, 73% of the hypotheses were confirmed (Table 5).

**Table 5:** Hypotheses for PROMIS-29 construct validity

PROMIS-29	Spearman’s rank correlations		Known-groups differences	Hypotheses confirmed (%)
	r ≥ 0.70	r ≤ 0.60		
Physical function	SF-12 PF SF-12 PCS	All other dissimilar constructs of legacy measures (n = 8)	Females will have lower physical function scores than males; Patients ages > 65 will have lower physical function scores than patients ages < 50; Patients with type A AD will have lower physical function scores than type B AD patients; Patients with CCI ≥ 3 will have lower physical function scores than patients with CCI < 2	76%
Anxiety	GAD-2 SF-12 MCS	All other dissimilar constructs of legacy measures (n = 8)	Females will have higher anxiety scores than males; Patients with type A AD will have higher anxiety scores than Type B AD patients	82%
Depression	PHQ-2 SF-12 MCS	All other dissimilar constructs of legacy measures (n = 8)	Females will have higher depression scores than males;	73%
Fatigue	SF-12 vitality	All other dissimilar constructs of legacy measures (n = 8)	Females will have higher fatigue scores than males;	82%
	SSS-8 tiredness		Patients with CCI ≥ 3 will have higher fatigue scores than patients with CCI < 2	
Sleep disturbance	SSS-8 sleep	All other dissimilar constructs of legacy measures (n = 9)	Females will have higher sleep disturbance scores than males	91%
Social roles	SF-12 SF	All other dissimilar constructs of legacy measures (n = 9)	Females will have lower social roles scores than males; Patients ages > 65 will have lower social roles scores than patients ages < 50; Patients with type A AD will have lower social roles scores than type B AD patients; Patients with CCI ≥ 3 will have lower social roles scores than patients with CCI < 2	86%
Pain interference	SSS-8 pain	All other dissimilar constructs of legacy measures (n = 9)	Females will have higher pain interference scores than males	91%

### Reliability

Internal consistency was excellent for all seven PROMIS-29 subscales, with Cronbach's alphas ranging from 0.92 (sleep disturbance) to 0.99 (pain interference). See Table 2.

## Discussion

To our knowledge, this study represents the first psychometric test of the Chinese version of the PROMIS-29 profile among AD survivors. The results of this study provide evidence and extend previous findings of the psychometric properties of the PROMIS-29. Overall, the PROMIS-29 has sufficient validity and reliability and is very efficient when used for measuring health status, including physical, mental, and social aspects, in this population. Current studies on PROs or QOL in people with AD usually use the SF-36 as the assessment tool. However, one limitation of the SF-36 is that it cannot directly measure important symptoms in AD patients, such as sleep problems, fatigue, anxiety and depression [10–12]. From this perspective, the PROMIS-29 may be more suitable for this patient group.

Evidence of floor and ceiling effects has been observed in some PROMIS-29 domains, similar to those of legacy measures. This result has also been noted in earlier PROMIS validation projects [20, 21, 42]. In addition, such effects for PROMIS-29 domains are comparatively minor compared to legacy scales. The relatively large ratios of people with pain interference, depression, fatigue, and anxiety scores at the scale floor are in agreement with the skewed distribution of these domains. This finding could have been generated by the length of the PROMIS-29 profile, with only four items in each domain. Minimizing the length of the questionnaires may have decreased the burden on respondents but at the same time caused insufficient measurement precision and breadth [43]. Another possible explanation for this might be that all AD patients in this sample demonstrated a stable condition with a mean duration of 11 months after surgical intervention. Accordingly, they were more likely to report a lack of such symptoms, especially pain. For AD patients, sharp pain was the most common onset symptom. Further exploration including AD patients during the acute phase is suggested to thoroughly evaluate the discrimination at lower levels of pain interference. The ceiling effect on physical function (33%) was markedly lower than those of other studies using a general population sample, for example, 72% of Coste et al. [44] and 71% of Garratt et al. [16]. It may still reflect a restriction on responsiveness; nevertheless, it would not be problematic when identifying those with poor physical performance. Such limitations may not exist in a future sample including more patients at an active stage of the disease.In addition, PROMIS can be administered as computerized adaptive tests (CATs), which allow dynamic selection of items based on the respondent’s prior answers, offering benefits such as improved precision and low question burden. Longer PROMIS short forms or CATs are also recommended to further explore these issues.

In general, the original PROMIS-29 seven-factor structure was supported without any modification in CFA. Meanwhile, nearly all single-factor structures fit the data well using the CFI and SRMR indices. RMSEA values were higher than expected for all but one scale. Similar findings were aligned with Rimehaug et al. [40] and Cook et al. [41]. This result may be attributed to the skewed data distribution in this sample [41]. Model fit was not ideal for sleep disturbance, which was also found in previous studies [40, 45]. In this scale, questions regarding “sleep quality” and “refreshment of sleep” may have some shared measurement error. Similar findings were also observed by Kang et al. [19] in an exploratory factor analysis using a sample of South Korean patients with chronic pulmonary diseases. Respondents’ bias in interpretations of items or an overlap between these two items may have caused these correlated errors. In addition, Cronbach’s alphas in excess of 0.9 for all domains, to some extent, indicate some potential item redundancy. In fact, some participants complained that the questionnaire involved repetitive questions. Taking the above points into consideration, a pragmatic solution to address such redundancy may be helpful for the future use of PROMIS-29 in this sample.

With respect to convergent validity, the PROMIS-29 domains showed adequate correlations with all corresponding legacy measures except SF-12 PCS and SF-12 MCS. Specifically, the correlation coefficient did not reach 0.7 between PROMIS-29 physical function and SF-12 PCS and between PROMIS-29 anxiety or depression and SF-12 MCS. The same results have been observed in other studies [46, 47]. Based on the literature [46], we speculated that the influence of patient-related factors on SF-12 scores may be one reason for this lack of association. Future longitudinal research with consideration of known patient-related factors may be helpful to further examine the relationship between the PROMIS domains and SF-12 PCS and MCS in this population. As initially assumed for discriminant validity, ≥ 75% of correlation coefficients between dissimilar PROMIS domains and legacy PRO subscales were lower than 0.6. Although relatively brief, these measures revealed expected results of psychometric properties of PROMIS-29 consistent to those previously presented by McMullen et al. [20], Quach et al. [48] and Kroenke et al. [49]. All the while, SF-36 seems to be more frequently used as a comparison to measure similar concepts in former PROMIS validation studies [42, 44, 49]. The validity and brief legacy questionnaires in the current study were based on a pragmatic choice, given the context of data collection via phone interviews.

Within the PROMIS-29, correlations between anxiety and depression and between physical function and the ability to participate in social roles and activities were relatively high (r = 0.76, r = 0.75, respectively). This is not uncommon, and similar results were also found in other samples, such as people with burn injuries [20], the general population [40] and cancer patients [45]. Such strong correlations indicate overlap between dimensions and may suggest the existence of potential high-order factors. Therefore, future comprehensive validation of the PROMIS-29 is clearly warranted, and cognitive interviews will be helpful.

The satisfactory discriminant validity of the PROMIS-29 was supported based on the results of known-group comparisons. An assumption between genders was that women may show worse physical function performance than men [50]. This was indeed confirmed. Then, it was hypothesized that females would have higher depression and anxiety scores according to previous literature. However, although a trend was seen in the expected direction, there was no statistical significance. These results were inconsistent with other observations [20, 21] and may partly be explained by the fairly low numbers of females included in the present study. In contrast to younger patients, older individuals presented lower physical function scores, as expected, which was concordant with the findings of other studies [20, 21, 51]. Finally, the anxiety, fatigue, physical function, and ability to participate in social roles and activities scores can distinguish between groups with known differences in the type of dissection and comorbidity burden measured by the CCI as initially assumed.

### Limitation

There are several limitations in this study that should be noted. First, the data in this study were obtained from a single center in central China, and the findings should be generalized with caution to other settings and populations. Additionally, the number of female participants was too small, which may have led to some bias. A possible explanation for these sample characteristics is that women have a lower incidence and a higher mortality of this aortic disease than men [52].

Second, test–retest analysis was not conducted in the present study as a result of time limits and limited resources. Additional research that included assessment about test–retest reliability would further support the temporal stability of this instrument.

Third, due to the cross-sectional design, the responsiveness and interpretability to change in different clinical statuses were not evaluated in this study. Future longitudinal investigation will be required to help determine the ability of the PROMIS-29 to detect changes in QOL and establish minimal important differences.

## Conclusions

This study found evidence for acceptable structural validity, construct validity and internal consistency of the PROMIS-29 profile in a sample of AD patients. It has been established that these short scales can be applied to AD survivors by researchers or clinicians, measuring outcomes after surgery and identifying those with worse health status. However, the sensitivity to change of the PROMIS-29 remains unclear for this specific population and needs to be established before its use in longitudinal studies. At the same time, further qualitative research is recommended to determine supplementation of AD-relevant items that may not be contained within PROMIS domains. Using generic combined with disease-specific measures may be beneficial to obtain a more comprehensive picture of patient-reported health outcomes.

## Data Availability

The datasets used and/or analyzed during the current study are available from the corresponding author on reasonable request.

## Abbreviations

PROMIS-29: The patient-reported outcomes measurement information system 29-item profile
AD: Aortic dissection
SF-12: Short form-12 health survey
SSS-8: 8-Item somatic symptom scale
GAD-2: Generalized anxiety disorder-2
PHQ-2: Patient health questionnaire-2
CFA: Confirmatory factor analysis
PROs: Patient reported outcomes
SD: Standard deviation
QOL: Quality of life
PF: Physical functioning
SF: Social functioning
PCS: Physical component summary
MCS: Mental component summary
PHQ-15: Patient health questionnaire-15
DSM-5: Fifth diagnostic and statistical manual of mental disorders
CCI: Charlson comorbidity index
CFI: Comparative fit index
SRMR: Standardized root-mean-squared residual
RMSEA: Root mean square error of approximation
AIC: Akaike’s information criterion
